# Supplementary material for: Implementation pilot study of community self-testing for COVID-19 among employees of manufacturing industries and their household members in 2022 to 2023
Source: PLOS Glob Public Health. 2024 Jun 5;4(6):e0003269. doi: 10.1371/journal.pgph.0003269 (PMC11152268; doi:10.1371/journal.pgph.0003269)
Supplement: S1 Annex — (DOCX) [file pgph.0003269.s001.docx]

**Supporting information**

**S1 Annex: Structured survey administered at baseline**

**Survey at enrolment (day 1)**

**INFORMATION TO PARTICIPANTS**

**This questionnaire will be anonymised before being analysed, and your name will never appear in the database.** **Your answers will be used to help us to better understand COVID-19 self-testing in Malaysia.**

**Demographics**

1. **Study site** [ list of site names]
2. **Study ID** [ MCS01 - _ _ - _ _ _ _ ]
3. **What is your age?** [list of numbers from 18–20; 21–25, in groups of 5 until 76 or more]
4. **What is your gender?**

- Male
- Female

Other, please specify: __________

- Prefer not to say

1. **What is your nationality?**
   - Malaysian
   - Other, please specify: __________
2. **Which ethnic group do you belong to?**
   - Malay
   - Chinese
   - Indian
   - Other, please specify: __________
3. **What is your native/primary language?**

- Bahasa Malaysia
- Mandarin
- Tamil
- English
- Other, please specify:__________

1. **What is your current employment status?**

- Employed full-time
- Employed part-time
- Other, please specify:__________

1. **What is the highest level of education that you have completed?**

- None
- Primary
- Secondary
- Post-secondary (Certificate, Foundation, Matriculation, STPM, Diploma)
- University (Bachelor’s degree, Master’s degree, PhD)

****Note for subsequent questions**** *Definition of people living in your household: Any person living with you in the same house/apartment/dormitory/room during the study*

1. **How many people live in your household,** **NOT including yourself?** [list of numbers from 0 to 15, more than 15]
2. [If Q10 is different to “0”] **How many people in your household were employed/paid for work in the past 3 months, NOT including yourself?** [list of numbers from 0 to 15, more than 15]
3. **How many children (under 12 years of age) are there in your household**? [list of numbers from 0 to 15, more than 15]
4. **How many adolescents (aged 12–17 years) are there in your household?** [list of numbers from 0 to 15, more than 15]
5. **Do you own a smartphone?** Single choice [Yes, No]

**Experiences with COVID-19**

1. **Are you vaccinated against COVID-19?**

- Yes, I have received one dose
- Yes, I have received two doses
- Yes, I have received three or more doses
- No, I have not received any doses

1. **Have you been diagnosed with COVID-19 before?**

- Yes
  - No
  - Uncertain

*If diagnosed with COVID-19 before,*

**16a. How severe was your experience with COVID-19? Select all that apply.**

- Asymptomatic (category 1)
- Mild to moderate symptoms (category 2)
- Severe symptoms (lung infection, respiratory distress, required hospital admission) (category 3–5)

1. Have any of your family members or close friends been diagnosed with COVID-19 before?

- Yes
- No
- Don’t know/Don’t remember

*If any of your family members or close friends have been diagnosed with COVID-19 before,*

**17a. If more than one family member or close friend had COVID-19, please answer, for the one who was most affected, how were their symptoms?**

- Asymptomatic (category 1)
- Mild to moderate symptoms (category 2)
- Severe symptoms (lung infection, respiratory distress, required hospital admission) (category 3–5)
- Don’t know

**17b. Have any of your family members or close friends passed away due to COVID-19?**

- Yes
- No
- Don’t know

**Perceptions and satisfaction with COVID-19 self-testing**

1. **How much do you agree with the following sentence “Currently, I am worried about the COVID-19 situation”?**

Likert scale [Strongly agree, agree, neutral, disagree, strongly disagree]

1. **How much do you agree with the following sentence: “I will perform COVID-19 self-tests when needed (if I have symptoms, if I am a close contact of a case, or other reasons), as part of the study”?**

Likert scale [Strongly agree, agree, neutral, disagree, strongly disagree]

1. **How much do you agree with the following sentence: “I will report COVID-19 self-tests results to my employer/MySejahtera after performing my self-test”?**

Likert scale [Strongly agree, agree, neutral, disagree, strongly disagree]

1. **How much do you agree with the following sentence: “I understand the benefits of self-testing for COVID-19”?**

Likert scale [Strongly agree, agree, neutral, disagree, strongly disagree]

1. **What benefits do you see in self-testing at home and having self-tests available for household members? Select all that apply.**
   - Self-test at home for my convenience
   - Self-test at home to reduce potential contacts
   - Provide a self-test for my household members
   - I don’t see any benefits compared with other COVID-19 diagnostics
   - Other, please specify: ____________________

**Knowledge**

1. **Where should you take a nasal swab sample for COVID-19 self-testing?**
   - In one nostril, no more than 2.5-cm deep
   - In two nostrils, no more than 2.5-cm deep
   - In two nostrils, more than 2.5-cm deep
   - In two nostrils, it does not matter how deep, but move the swab in circles
   - In the mouth and one or two nostrils
2. **What does a positive COVID-19 self-test result mean?**
   - I am not infected with COVID-19
   - I had COVID-19 in the past
   - I have a high likelihood of developing severe symptoms
   - I am infected with COVID-19
   - I don’t know
3. **What do you need to do if you test positive following a COVID-19 self-test? Select all that apply.**
   - Check the updated national guidelines
   - Self-isolate, as much as possible
   - Immediately report results to my employer
   - Immediately report results to MySejahtera
   - Call my close contacts
   - Wear a mask to work and maintain hygiene measures
4. **What does a faint line at the T line and a clear line at the C line mean in the COVID-19 self-test?**

- I have COVID-19 disease and can infect others
- I need to repeat the COVID-19 self-test
- It is a false-positive result
- I don’t have COVID-19
- I have COVID-19 but cannot infect others

1. **If your self-test results is negative for COVID-19 but you have symptoms, what should you do? Select all that apply.**

- Repeat the self-test immediately
- Repeat a self-test on the third day of symptoms
- Check if my symptoms worsen and consult a doctor
- Go to work and wear a mask, I don’t need to test again
- I don’t know
