# Supplementary material for: Implementation pilot study of community self-testing for COVID-19 among employees of manufacturing industries and their household members in 2022 to 2023
Source: PLOS Glob Public Health. 2024 Jun 5;4(6):e0003269. doi: 10.1371/journal.pgph.0003269 (PMC11152268; doi:10.1371/journal.pgph.0003269)
Supplement: S2 Annex — (DOCX) [file pgph.0003269.s002.docx]

**Supporting information**

**S2 Annex: Structured survey administered at end-point**

**INFORMATION TO PARTICIPANTS**

**This questionnaire will be anonymised before being analysed and your name will never appear in the database.** **Your answers will be used to help us better understand COVID-19 self-testing in Malaysia.**

1. **Study site [**list of sites]

1. **Study ID** [ MCS01 - _ _ - _ _ _ _ ]

**Experiences with COVID-19 self-testing**

1. **How many of the COVID-19 self-tests that you received from the study were damaged (could not be used)?** **Note: Invalid test results are not considered to be damaged tests.** [List from 0 to 15, more than 15, don’t know]

1. **How many COVID-19 self-tests did you use for yourself during the study?** [List from 0 to 15, more than 15, don’t know]

*If you did NOT perform a COVID-19 self-test as part of the study:*

**4a. What were the reasons?**

- I forgot I had the self-tests for use at home
- I did not have any COVID-19 symptoms or any close contact with a COVID-19-positive person
- I did not want to self-test
- I did not want to use the nasal swab for self-testing
- Other, please specify: ______________

*If you did perform a COVID-19 self-test as part of the study:*

**4b. Did you have any invalid results?**

- - Yes
  - No

**4c. Did you have any positive results?**

- - Yes
  - No

*If you tested positive for COVID-19 when using a self-test:*

**4c1. Select all of the responses that apply regarding the actions you took following your positive self-test result:**

- Self-isolated
- Told my close contacts
- Told my employer
- Reported to MySejahtera
- Continued to go to work
- Visited a healthcare clinic or hospital
- Checked updated local guidelines for COVID-19

**4c2. Did you report your self-test results via the study reporting form?**

- Yes
- No

**4c2.1 If you did not report your results via the study reporting form, what was the reason for this?**

- I forgot to report the results
- I did not know how to access the reporting form
- I did not want to report my results to the study
- I did not understand that I needed to report every time I self-tested
- I reported my results to my employer
- I reported my results to MySejahtera
- Other, please specify: ____________________

**4c3. How severe was your experience of COVID-19? Select all that apply.**

- Asymptomatic [Category 1]
- Mild to moderate symptoms [Category 2]
- Severe symptoms (lung infection, respiratory distress, required hospital admission) [Category 3–5]

**5. During the study, how many COVID-19 self-tests were used by your household members, relatives or friends?** [list from 0 to 15, more than 15, don’t know]

*If your household members, relatives or friends performed a COVID-19 self-test as part of the study:*

**6. How many of the self-tests from the study used by household members, relatives or friends gave an invalid result?** [list from 0 to 15, more than 15, don’t know]

**7. How many of your household members, relatives or friends performed a COVID-19 self-test?** [list of numbers from 0 to 15, more than 15, don’t know]

**8. How many of your household members, relatives or friends had a positive self-test result?** [list from 0 to 15, more than 15, don’t know]

*If your household members, relatives or friends had a positive self-test result as part of the study:*

**8.a. If more than one household member, relative or friend had a positive result, please answer for the person who was most affected; select all responses that apply regarding their/your actions:**

- They self-isolated
- They told their close contacts
- They told their employer
- I told my employer
- They reported to MySejahtera
- They continued to go to work
- They checked the updated local guidelines for COVID-19
- They visited a healthcare clinic or hospital
- I don’t know

**8.b. If more than one household member, relative or friend had a positive self-test result, please answer for the person who was most affected, how were their symptoms?**

- Asymptomatic [Category 1]
- Mild to moderate symptoms [Category 2]
- Severe symptoms (lung infection, respiratory distress, required hospital admission) [Category 3–5]
- Don’t know

**Perceptions and satisfaction with COVID-19 self-testing**

1. **How much do you agree with the following sentence “Currently, I am worried about the COVID-19 situation”?**

Likert scale [Strongly agree, agree, neutral, disagree, strongly disagree]

1. **How much do you agree with the following sentence: “I performed COVID-19 self-tests when needed (if I had symptoms, if I was a close contact of a case, or other reasons), as part of the study”?**

Likert scale [Strongly agree, agree, neutral, disagree, strongly disagree]

1. **How much do you agree with the following sentence: “I reported COVID-19 self-tests results to my employer/MySejahtera after performing my self-tests”?**

Likert scale [Strongly agree, agree, neutral, disagree, strongly disagree]

1. **How much do you agree with the following sentence: “I understand the benefits of self-testing for COVID-19”?**

Likert scale [Strongly agree, agree, neutral, disagree, strongly disagree]

1. **What benefits do you see in self-testing at home and having self-tests available at home for household members? Select all that apply.**

- Self-test at home for my convenience
- Self-test at home to reduce potential contacts
- Provide a self-test for my household members
- I don’t see any benefits compared with other COVID-19 diagnostics
- Other, please specify: ____________________

**Knowledge**

1. **Where should you take a nasal swab sample for COVID-19 self-testing?**

- In one nostril, no more than 2.5-cm deep
- In two nostrils, no more than 2.5-cm deep
- In two nostrils, more than 2.5-cm deep
- In two nostrils, it does not matter how deep, but move the swab in circles
- In the mouth and one or two nostrils

1. **What does a positive COVID-19 self-test result mean?**

- I am not infected with COVID-19
- I had COVID-19 in the past
- I have a high likelihood of developing severe symptoms
- I am infected with COVID-19
- I don’t know

1. **What do you need to do if you test positive when using a COVID-19 self-test? Select all that apply.**

- Check the updated national guidelines
- Self-isolate as much as possible
- Report results immediately to my employer
- Report results immediately to MySejahtera
- Call my close contacts
- Go to work with a mask and maintain hygiene measures

1. **What does a faint line at the T line and a clear line at the C line mean in the COVID-19 self-test?**

- I have COVID-19 disease and can infect others
- I need to repeat the COVID-19 self-test
- It is a false-positive result
- I don’t have COVID-19
- I have COVID-19 but cannot infect others

1. **If you are negative for COVID-19 but have symptoms, what should you do? Select all that apply.**

- Repeat the self-test immediately
- Repeat a self-test on the third day of symptoms
- Check if my symptoms worsen and consult a doctor
- Go to work and wear a mask, I don’t need to test again
- I don’t know
