# Supplementary material for: Implementation pilot study of community self-testing for COVID-19 among employees of manufacturing industries and their household members in 2022 to 2023
Source: PLOS Glob Public Health. 2024 Jun 5;4(6):e0003269. doi: 10.1371/journal.pgph.0003269 (PMC11152268; doi:10.1371/journal.pgph.0003269)
Supplement: S3 Annex — (DOCX) [file pgph.0003269.s003.docx]

**Supporting information**

**S3 Annex: Semi-structured interview guide**

| **Date:**  **Interviewer:**  **Site:**  **#SSI:**  **Guide version:**  **Participant pilot ID number:** |
| --- |
| **Opening statement:**  Thank you for agreeing to take part in this interview today. We will be discussing your experiences with COVID-19 self-testing and your views on how self-testing should be provided.  **Topics to be explored:**  The topics that we will be discussing today include your perceptions and experiences of using self-tests, potential barriers and factors that could facilitate/motivate you to perform self-testing, and behaviours after receiving a positive result. |
| **Demographic data:** |
| **Perceptions and experiences of COVID-19 self-testing**   - Previous experience with COVID-19 in the family - Previous experience with self-tests? Have you self-tested before the pilot? What type of self-tests? - How would you describe your experience in the pilot? Did you perform any self-tests? Did you experience any difficulties while performing the self-test? How was the experience? What were the results (positive, negative, invalid)? What action did you take after taking a test? (self-isolation, reported the result via MySejahtera, reported the result to your employer, disclosed the result to your contacts etc.) - Did you report your result? If not, what was the reason? (could not find the QR code, did not know there was a reporting form, did not have time, etc.) - Household members’ experience in this pilot. Did any of your household members use a self-test? What were the results? Was it easy/difficult? - What were your reasons for using a self-test? - What do you think are the main reasons why people choose to use COVID-19 self-testing? Under which circumstances? - Did your perception change after using a self-test? - Do you think that your satisfaction with self-testing changed from the beginning of the pilot until now? In what sense? (e.g. your confidence in performing the test, recommending it to others etc.) - How do you feel now, going to work now, compared with months ago (safer, stressed, overwhelmed, calm etc)? - What are your colleague’s experiences of self-testing? Did any of them use a self-test? Did you discuss the pilot study with your colleagues? - Do you think other people (among the general population) would be interested in self-testing? Why or why not? - What do you think were the main advantages of this pilot study? Were there any disadvantages? - Would you use a COVID-19 self-test again in the future (not for the workplace)? Why or why not? - What do you think about the use of self-tests for other diseases? Would you find it useful to have self-tests available to test for flu, dengue or other diseases? Would you use these self-tests if they were available?   *Based on end-point survey respondents*   - We noticed that you reported that XX COVID-19 self-tests you received from the pilot study were damaged and could not be used. Could you please describe how these tests were damaged or could not be used? - Why do you think they were damaged/could not be used? - We noticed that you reported that you had XX invalid COVID-19 self-test results. Could you please share with us why you think the results were invalid? - Do you know the difference between a damaged self-test and invalid self-test results? - We noticed that in your response, you mentioned you reported your self-test results via the pilot study reporting form. Did you scan the QR code/click the URL to access the reporting link on Google Forms? Did you report elsewhere, such as via MySejahtera, to your employer etc? |
| **Other questions:**  Do you have any questions/concerns/comments that we haven’t addressed and that you would like to share? Do you have anything else to add? |
| *Thank you for your time and participation. We have learnt a lot from our discussion here today and we hope the time has also been useful to you.* |
