# Supplementary material for: Implementation pilot study of community self-testing for COVID-19 among employees of manufacturing industries and their household members in 2022 to 2023
Source: PLOS Glob Public Health. 2024 Jun 5;4(6):e0003269. doi: 10.1371/journal.pgph.0003269 (PMC11152268; doi:10.1371/journal.pgph.0003269)
Supplement: S4 Annex — (DOCX) [file pgph.0003269.s004.docx]

**Supporting information**

**S4 Annex: Focus group discussion guide**

| **Facilitator’s full name:**  **Facilitator’s assistants’ (note-takers) full names:** |
| --- |
| [INFORMED CONSENT + sociodemographic information]  Good morning everyone and thank you very much for agreeing to take part in this focus group discussion today.  My name is [XXX], I am a researcher working at [xxx] and I will be moderating today’s discussion. Today, my colleagues [xxx and xxx] will just be observing and taking notes.  The objective of this discussion is to explore your experiences and any difficulties with the COVID-19 self-testing project in the factory where you work.  The topics that we will be discussing today include:   - Background information about how COVID-19 testing was performed in your workplace, if there were any protocols in place before the project, and what happened if there were employees with symptoms. - Operational issues about the implementation of the COVID-19 self-testing project. - General perceptions, experiences, and satisfaction with COVID-19 self-testing and with the project itself. - Potential self-testing models for other diseases. - The opportunity to comment on anything or to discuss any other topic, that we have not asked about.   So, here are some basic norms and rules about how this discussion will go:   1. I will be **asking some questions to the group, and anyone can answer.** 2. Each question could be answered **by one, two or three individuals or even by all participants**. You do not need to follow any specific order to answer, just **feel free to raise your hand and speak.** 3. Also, **you can interact with the others.** Try to respect others’ time while speaking, do not interrupt them, but feel free to talk when the other person has finished. 4. Something important to remember is that there **are no right or wrong responses**, this is not a test, and all of your responses are valid! This is a free discussion!    1. So, even if you disagree with somebody, please respect their opinion.    2. It is very valuable for us to hear about the different opinions, perceptions and experiences you all have.    3. So again, don’t be afraid to speak and don’t be afraid to give a different opinion from somebody else. 5. We would prefer to have this discussion without any interruptions, but if somebody needs to stop for something, please do let me know, and we’ll consider whether to stop and wait or to continue, depending on how the discussion is going. 6. The whole discussion will last for approximately one to one and a half hours. 7. I will try not to mention your names, but do not worry if I or anyone else mentions your names, as we will only report the results of these discussions in an aggregate form. We will never disclose who said what. 8. Finally, we would like to inform you that we might use some of your “quotes”, “the sentences that you may say” for our results and our online materials (on the website); again, this will be anonymously. Please let me know, now or at the end of the discussion, if any of you have any problem with us publishing your quotes.   Do you have any questions or doubts that I can address before starting the recording?  With your permission, I will start the recording of the audio. |
| **Good morning, today is [___**day**__] of [____**month**___] of 2023, and we are [**at (location)/conducting an online FGD with representatives from [________________] site**].**  **[5-10 min] General questions**   1. We would like to start with your experiences of testing for COVID-19 before the project. Could you tell us, how did employees test for COVID-19 before our project study? (e.g. PCR, professional antigen test, serology, self-test, or assisted self-test)    - How? (PCR, self-testing, professional antigen test, other)    - When? (when they had symptoms, were a close contact of a case, or any other circumstances, according to the national guidelines; routine weekly/monthly monitoring other)    - *How was your experience? Did you like it or not? Why?*   **[15-25 min] Great! From now until the end of the discussion, we are going to talk about our COVID-19 self-testing project implemented in the factory.**  **Operational issues around the implementation of the COVID-19 self-testing project**   1. **Did any of you use a COVID-19 elf-test during the project?**    - Why? Could you share the reasons why you used the self-test?    - How about your household members?    - Did you report the result (negative, invalid or positive) through the survey link?      - If yes, how was the experience? Was it easy/difficult/quick/time-consuming?      - If no, could you please explain why not? 2. **Do you think that employees used the self-test? Why?** à [IMPORTANT to have the maximum number of responses] 3. **Do you think that employees used the self-test but did not report their results via the survey link? Why?** à [IMPORTANT to have the maximum number of responses] 4. [To be prepared before each FGD: “show specific results from the site”] Only a few participants have reported their COVID-19 self-test results to us. In your opinion, **how would you explain this? What could be happening?**    - Do you think that people **do not feel the need to self-test for COVID-19?**       - Or do they not have the **awareness**?    - Do you think that people **prefer to test using other methods**?      - Or do they prefer to test at a **healthcare centre/work**?    - Do you think that people did not want to share their results/forgot/did not know how or when to do it? 5. Do you think that this COVID-19 self-testing project brought any benefit to the company, to employees, or to their household members/the community? What were these benefits? 6. Do you know what the expiry date is of the COVID-19 self-testing kits we provided?    - What would you do if you realised the test kits were due to expire soon?     **[5-10 min] PARTICIPATORY ACTION: Find potential solutions.**  Next, we will be brainstorming together to find any potential problems or difficulties encountered and identify potential solutions.  **What were the main difficulties of implementing the COVID-19 self-testing project?**   - - For example, regarding training, logistics, managing the company, data collection through online surveys, use of nasal self-testing?   - Were those difficulties addressed?     - How? How did it go? Could another way have worked better?     - If not, why not?   - Could you share some tips about the implementation of this project at your site that could be useful for other workplaces?   **What do you think were the main problems** and how could we solve them? **How could your site help to address them?**    **[5-10 min] Self-testing models**   1. Based on your knowledge and experience now, are there any other health programmes that you would like to integrate in your factory?    - Which health programmes?    - Any programmes that involve self-testing?    - For which diseases? Only if they do not mention any: flu, malaria, dengue, chikungunya, zika, tuberculosis? 2. **Which materials (online or printed) would you like to have to help you implement another self-testing strategy/health programme in the factory in the future?**    - Would you find it helpful to have an online or printed “tool kit”, with steps to follow to implement another self-testing strategy for other infectious diseases? 3. Do you think that other factories would find it useful to have this “tool kit”?    - Which type of factories?    - Those that had less access to COVID-19 testing in the community? The ones with the worst/best working conditions? More/less digitalised companies? Large/small companies in terms of the size of their workforce?   **[5-10 min] Perceptions, experiences and satisfaction with COVID-19 self-testing in the factory**   1. How was your general satisfaction with the project? 2. How was your experience with the project? (Anything you would like to share?)    - Did you experience any other difficulties during these months? What were they? 3. **Did participants at your site report any positive results?**    - **What did they do?**    - **What did the factory recommend they do? (self-isolate, take paid medical leave, wear a mask at work)** 4. Did participants report any invalid result?    - If so, what did they do? (repeat a self-test, report via MySejahtera, etc.) |
| **[5-10 min] Other**   1. Do you have any other questions, comments, or concerns that we have not addressed today that you would like to share? Anything else to add? |
| Thank you for your time and participation. We have learnt a lot from our discussion here today, and we hope the time has also been useful to you. |
