# Supplementary material for: Implementation pilot study of community self-testing for COVID-19 among employees of manufacturing industries and their household members in 2022 to 2023
Source: PLOS Glob Public Health. 2024 Jun 5;4(6):e0003269. doi: 10.1371/journal.pgph.0003269 (PMC11152268; doi:10.1371/journal.pgph.0003269)
Supplement: S7 Annex — (DOCX) [file pgph.0003269.s007.docx]

**Supporting information**

**S7 Annex: Reported uptake of COVID-19 self-tests**

| **Aspect** | **n/N (%)** |
| --- | --- |
| Uptake of self-testing by participants  No/uncertain  Yes | 278/674 (41.2)  396/674 (58.8) |
| Results of self-testing by participants  Positive  Negative  Invalid | 40/396 (10.1)  329/396 (83.1)  27/396 (6.8) |
| Uptake of self-testing by household members  No/uncertain  Yes | 296/671 (44.1)  375/671 (55.9) |
| Having at least one household member with a positive result  No  Yes | 275/375 (73.3)  100 (26.7) |
