# Supplementary material for: Implementation pilot study of community self-testing for COVID-19 among employees of manufacturing industries and their household members in 2022 to 2023
Source: PLOS Glob Public Health. 2024 Jun 5;4(6):e0003269. doi: 10.1371/journal.pgph.0003269 (PMC11152268; doi:10.1371/journal.pgph.0003269)
Supplement: S8 Annex — (DOCX) [file pgph.0003269.s008.docx]

**Supporting information**

**S8 Annex: Simple logistic regression analysis of potential associations between participants’ characteristics and their uptake of COVID-19 self-testing.**

| **Characteristic** | **Did not report self-testing use, n (%)** | **Reported self-testing use, n (%)** | **Crude odds ratio (95% CI)** | **p-value** |
| --- | --- | --- | --- | --- |
| Site  Production-based  Office-based | 253 (41.6)  7 (25.0) | 355 (58.4)  21 (75.0) | 1  2.14 (0.90, 5.11) | -  0.087 |
| Age  ≤30  31–40  41–50  >50 | 39 (28.7)  29 (33.3)  110 (44.2)  82 (50.0) | 97 (71.3)  58 (66.7)  139 (55.8)  82 (50.0) | 2.49 (1.54, 4.03)  2.00 (1.17, 3.44)  1.26 (0.85, 1.88)  1 | **<0.001**  0.012  0.246  - |
| Gender  Male  Female | 79 (35.6)  181 (43.7) | 143 (64.4)  233 (56.3) | 1.41 (1.00, 1.97)  1 | **0.047**  - |
| Country of origin  Malaysia  Not Malaysia | 256 (40.9)  4 (40.0) | 370 (59.1)  6 (60.0) | 1  1.04 (0.29, 3.72) | -  0.954 |
| Employment status  Employed full-time  Employed part-time | 253 (40.7)  7 (46.7) | 36 (59.3)  8 (53.3) | 1.27 (0.46, 3.55)  1 | 0.645  - |
| Education level  None/primary  Secondary  Post-secondary  Tertiary | 4 (50.0)  142 (42.4)  73 (38.8)  41 (39.0) | 4 (50.0)  193 (57.6)  115 (61.2)  64 (61.0) | 1  1.36 (0.33, 5.53)  1.58 (0.38, 6.50)  1.56 (0.37, 6.69) | -  0.668  0.529  0.545 |
| Number of household members  0  1–2  3–4  ≥5 | 2 (25.0)  54 (48.2)  100 (38.0)  104 (41.4) | 6 (75.0)  58 (51.8)  163 (62.0)  149 (58.9) | 2.09 (0.41, 10.58)  0.75 (0.48, 1.17)  1.14 (0.80, 1.62)  1 | 0.371  0.207  0.474  - |
| Number of household members in employment in the past three months  0  1–2  3–4  ≥5 | 42 (37.8)  160 (41.0)  46 (40.4)  12 (57.1) | 69 (62.6)  230 (59.0)  68 (59.6)  9 (42.9) | 2.19 (0.85, 5.64)  1.92 (0.79, 4.66)  1.97 (0.77, 5.06)  1 | 0.104  0.151  0.158  - |
| Number of adolescents aged 12 to 17 years old)  0  1–2  3–4  ≥5 | 139 (40.4)  108 (42.0)  12 (36.4)  1 (50.0) | 205 (59.6)  149 (58.0)  21 (63.6)  1 (50.0) | 1.48 (0.91, 23.78)  1.38 (0.09, 22.30)  1.75 (0.10, 30.59) 1 | 0.784  0.821  0.701  - |
| Number of children (aged < 12 years old)  0  1–2  3–4  ≥5 | 162 (43.0)  85 (39.5)  12 (30.8)  1 (20.0) | 215 (57.0)  130 (60.5)  27 (69.2)  4 (80.0) | 1  1.15 (0.82, 1.62)  1.70 (0.83, 3.44)  3.01 (0.33, 27.22) | -  0.415  0.145  0.326 |
| Ownership of a smartphone  No  Yes | 8 (61.5)  252 (40.4) | 5 (38.5)  371 (59.6) | 1  2.35 (0.76, 7.28) | -  0.137 |
| Doses of COVID-19 vaccine  Two or fewer  Three or more | 40 (32.5)  220 (42.9) | 83 (67.5)  293 (57.1) | 1.56 (1.03, 2.36)  1 | **0.037**  - |
| History and severity of COVID-19 diagnosis  None/uncertain status or  severity  Yes, Category 1  Yes, Category 2  Yes, Category 3–5 | 113 (42.3)  21 (42.0)  119 (39.0)  7 (50.0) | 154 (57.7)  29 (58.0)  186 (61.0)  7 (50.0) | 1.36 (0.47, 3.96)  1.38 (0.42, 4.53)  1.56 (0.54, 4.57) 1 | 0.573  0.595  0.414  - |
| History and severity of COVID-19 diagnosis in their most affected family member/close friend, n (%)  None/uncertain status or  severity  Yes, Category 1  Yes, Category 2  Yes, Category 3–5  Yes, deceased | 77 (40.1)  24 (40.7)  119 (41.2)  6 (33.3)  34 (43.6) | 115 (59.9)  35 (59.3)  170 (58.8)  12 (66.7)  44 (56.4) | 1.15 (0.68, 1.97)  1.13 (0.57, 2.24)  1.10 (0.67, 1.83)  1.55 (0.53, 4.54)  1 | 0.598  0.733  0.701  0.428  - |
| Worried about COVID-19  Strongly disagree (1)  Disagree (2)  Neutral (3)  Agree (4)  Strongly agree (5) | 10 (35.7)  17 (40.5)  63 (41.2)  76 (44.7)  94 (38.7) | 18 (64.3)  25 (59.5)  90 (58.8)  94 (55.3)  149 (61.2) | 1.14 (0.50, 2.57)  0.93 (0.48, 1.81)  0.90 (0.60, 1.36)  0.78 (0.52, 1.16)  1 | 0.760  0.826  0.621  0.221  - |
| Willing to perform self-testing  Strongly disagree (1)  Disagree (2)  Neutral (3)  Agree (4)  Strongly agree (5) | 11 (55.0)  7 (43.8)  40 (42.6)  71 (45.5)  131 (37.4) | 9 (45.0)  9 (56.3)  54 (57.4)  85 (54.5)  219 (62.6) | 1  1.57 (0.42, 5.90)  1.65 (0.63, 4.36)  1.46 (0.57, 3.73)  2.04 (0.83, 5.06) | -  0.503  0.312  0.425  0.123 |
| Willing to report their self-testing result  Strongly disagree (1)  Disagree (2)  Neutral (3)  Agree (4)  Strongly agree (5) | 5 (41.7)  5 (38.5)  32 (40.5)  59 (43.7)  159 (40.1) | 7 (58.3)  8 (61.5)  47 (59.5)  76 (56.3)  238 (59.9) | 1  1.14 (0.23, 5.67)  1.05 (0.31, 3.60)  0.92 (0.28, 3.05)  1.07 (0.33, 3.43) | -  0.870  0.939  0.892  0.910 |
| Understand the benefits of self-testing  Strongly disagree (1)  Disagree (2)  Neutral (3)  Agree (4)  Strongly agree (5) | 6 (50.0)  5 (50.0)  24 (33.8)  69 (46.3)  156 (39.6) | 6 (50.0)  5 (50.0)  47 (66.2)  80 (53.7)  238 (60.4) | 1  1.00 (0.19, 5.36)  1.96 (0.57, 6.73)  1.16 (0.36, 3.76)  1.53 (0.48, 4.82) | -  >0.99  0.286  0.805  0.471 |

Significant associations (p < 0.005) are highlighted in bold for easy identification; CI, confidence interval
