# Supplementary material for: Implementation pilot study of community self-testing for COVID-19 among employees of manufacturing industries and their household members in 2022 to 2023
Source: PLOS Glob Public Health. 2024 Jun 5;4(6):e0003269. doi: 10.1371/journal.pgph.0003269 (PMC11152268; doi:10.1371/journal.pgph.0003269)
Supplement: S9 Annex — (DOCX) [file pgph.0003269.s009.docx]

**Supporting information**

**S9 Annex: Simple logistic regression analysis of potential associations between participants’ characteristics and the uptake of self-testing among their household members.**

| **Characteristic** | **Not reported self-testing use, n (%)** | **Reported self-testing use, n (%)** | **Crude odds ratio (95% CI)** | **p-value** |
| --- | --- | --- | --- | --- |
| Site  Production-based  Office-based | 270 (44.6)  15 (53.6) | 335 (55.4)  13 (46.4) | 1.43 (0.67, 3.06) 1 | 0.355  - |
| Age  ≤30  31–40  41–50  >50 | 54 (39.7)  37 (42.5)  111 (44.9)  83 (50.9) | 82 (60.3)  30 (57.5)  136 (55.1)  80 (49.1) | 1.58 (0.99, 2.50)  1.40 (0.83, 2.37)  1.27 (0.86, 1.89)  1 | 0.053  0.207  0.236  - |
| Gender  Male  Female | 100 (45.2)  185 (44.9) | 121 (54.8)  227 (55.1) | 1  1.01 (0.73, 1.41) | -  0.934 |
| Country of origin  Malaysia  Not Malaysia | 284 (45.6)  1 (10.0) | 339 (54.4)  9 (90.0) | 1  7.54 (0.95, 59.87) | -  **0.056** |
| Employment status  Employed full-time  Employed part-time | 278 (45.0)  7 (46.7) | 340 (55.0)  8 (53.3) | 1.07 (0.38, 2.99)  1 | 0.897  - |
| Education level  None/primary  Secondary  Post-secondary  Tertiary | 5 (62.5)  147 (44.3)  80 (42.6)  53 (50.5) | 3 (37.5)  185 (55.7)  108 (57.4)  52 (49.5) | 1  2.10 (0.49, 8.92)  2.25 (0.52, 9.69)  1.64 (0.37, 7.20) | -  0.316  0.276  0.515 |
| Number of household members  0  1–2  3–4  ≥5 | 4 (50.0)  59 (52.7)  119 (45.6)  103 (40.9) | 4 (50.0)  53 (47.3)  142 (54.4)  149 (58.1) | 1  0.90 (0.21, 3.77)  1.19 (0.29, 4.87)  1.45 (0.35, 5.92) | -  0.884  0.806  0.607 |
| Number of household members in employment in the past three months  0  1–2  3–4  ≥5 | 52 (46.8)  167 (43.2)  53 (46.5)  13 (61.9) | 59 (53.2)  220 (56.8)  61 (53.5)  8 (38.1) | 1.84 (0.71, 4.80)  2.14 (0.87, 5.28)  1.87 (0.72, 4.86)  1 | 0.210  0.099  0.199  - |
| Number of adolescents (aged 12 to 17 years)  0  1–2  ≥3 | 161 (46.9)  113 (44.3)  11 (31.4) | 182 (53.1)  142 (55.7)  24 (68.6) | 1  1.11 (0.80, 1.54)  1.93 (0.92, 4.06) | -  0.524  0.083 |
| Number of children (aged <12 years)  0  1–2  3–4  ≥5 | 182 (48.4)  88 (41.3)  13 (33.3)  2 (40.0) | 194 (51.6)  125 (58.7)  26 (66.7)  3 (60.0) | 1  1.33 (0.95, 1.87)  1.88 (0.94, 3.76)  1.41 (0.23, 8.52) | -  0.097  0.076  0.710 |
| Ownership of a smartphone  No  Yes | 7 (53.8)  278 (44.8) | 6 (46.2)  342 (55.2) | 1  1.44 (0.48, 4.32) | -  0.520 |
| Doses of COVID-19 vaccine received  Two or fewer  Three or more | 41 (33.6)  244 (47.7) | 81 (66.4)  267 (52.3) | 1.81 (1.19, 2.73)  1 | **0.005**  - |
| History and severity of COVID-19 diagnosis  None/uncertain status or  severity  Yes, Category 1  Yes, Category 2  Yes, Category 3–5 | 125 (46.8)    19 (38.0)  137 (45.5)  4 (28.6) | 142 (53.2)    31 (62.0)  165 (54.6)  10 (71.4) | 1    1.44 (0.77, 2.67)  1.06 (0.76, 1.48)  2.20 (0.67, 7.19) | -    0.252  0.729  0.192 |
| History and severity of COVID-19 diagnosis in their most affected family member/close friend  None/uncertain status or  severity  Yes, Category 1  Yes, Category 2  Yes, Category 3–5  Yes, deceased | 82 (42.7)  27 (45.8)  136 (47.4)  4 (22.2)  36 (46.8) | 110 (57.3)  32 (54.2)  151 (52.6)  14 (77.8)  41 (53.2) | 1.18 (0.60, 2.00)  1.04 (0.53, 2.06)  0.98 (0.59, 1.61)  3.07 (0.93, 10.18)  1 | 0.546  0.909  0.921  0.066  - |
| Worried about COVID-19  Strongly disagree (1)  Disagree (2)  Neutral (3)  Agree (4)  Strongly agree (5) | 13 (46.4)  22 (53.7)  68 (44.7)  77 (45.6)  105 (43.2) | 15 (53.6)  19 (46.3)  84 (55.3)  92 (54.4)  138 (56.8) | 1  0.75 (0.29, 1.96)  1.07 (0.48, 2.40)  1.04 (0.46, 2.31)  1.14 (0.52, 2.50) | -  0.556  0.869  0.932  0.745 |
| Willing to perform self-testing  Strongly disagree (1)  Disagree (2)  Neutral (3)  Agree (4)  Strongly agree (5) | 11 (55.0)  8 (50.0)  43 (45.7)  75 (48.7)  148 (42.4) | 9 (45.0)  8 (50.0)  51 (54.3)  79 (51.3)  201 (57.6) | 1  1.22 (0.33, 4.57)  1.45 (0.55, 3.82)  1.29 (0.51, 3.28)  1.66 (0.67, 4.11) | -  0.765  0.453  0.597  0.273 |
| Willing to report their self-testing result  Strongly disagree (1)  Disagree (2)  Neutral (3)  Agree (4)  Strongly agree (5) | 6 (50.0)  6 (46.2)  37 (46.8)  65 (48.5)  171 (43.3) | 6 (50.0)  7 (53.8)  42 (53.2)  69 (51.5)  224 (56.7) | 1  1.17 (0.24, 5.62)  1.14 (0.34, 3.83)  1.06 (0.33, 3.46)  1.31 (0.42, 4.13) | -  0.848  0.838  0.921  0.645 |
| Understand the benefits of self-testing  Strongly disagree (1)  Disagree (2)  Neutral (3)  Agree (4)  Strongly agree (5) | 7 (58.3)  6 (60.0)  29 (40.8)  72 (49.0)  171 (43.5) | 5 (41.7)  4 (40.0)  42 (59.2)  75 (51.0)  222 (56.5) | 1  0.93 (0.17, 5.15)  2.03 (0.59, 7.02)  1.46 (0.44, 4.81)  1.82 (0.57, 5.83) | -  0.937  0.264  0.535  0.315 |

Significant (p < 0.005)/almost significant (p = 0.005); these associations are highlighted in bold for easy identification.
