# Supplementary material for: Implementation pilot study of community self-testing for COVID-19 among employees of manufacturing industries and their household members in 2022 to 2023
Source: PLOS Glob Public Health. 2024 Jun 5;4(6):e0003269. doi: 10.1371/journal.pgph.0003269 (PMC11152268; doi:10.1371/journal.pgph.0003269)
Supplement: S10 Annex — (DOCX) [file pgph.0003269.s010.docx]

**Supporting information**

**S10 Annex: Participants’ COVID-19 self-test results reported via reporting survey, overall**

| **Characteristic** | **n** | **%** |
| --- | --- | --- |
| **Type of workplace**  Production-based  Office-based | 21/39  18/39 | 53.8  46.2 |
| **Reason for self-test:**  Symptoms  Contact of a case  Routine monitoring  Attending a mass gathering event  Visiting family/friends with comorbidities  Holidays | 19/39  3/39  2/39  16/39  5/39  1/39 | 48.7  7.7  5.1  41.0  12.8  2.6 |
| **One positive result reported** | 2/39 | 5.1 |
| **Number of COVID-19 self-tests used by participants:**  1  2–3  ≥4 | 26/39  11/39  2/39 | 66.7  28.2  5.1 |
| **Number of COVID-19 self-tests used by household members:**  0  1  2–3  ≥4 | 2/30  5/30  14/30  9/30 | 6.7  16.7  46.7  30.0 |
