# Supplementary material for: Implementation pilot study of community self-testing for COVID-19 among employees of manufacturing industries and their household members in 2022 to 2023
Source: PLOS Glob Public Health. 2024 Jun 5;4(6):e0003269. doi: 10.1371/journal.pgph.0003269 (PMC11152268; doi:10.1371/journal.pgph.0003269)
Supplement: S11 Annex — (DOCX) [file pgph.0003269.s011.docx]

**Supporting information**

**S11 Annex: Sociodemographic characteristics of participants interviewed**

| Variable | Category | Semi-structured interviews  N=44 | Focus group discussions  N=14 |
| --- | --- | --- | --- |
|  | | **n (%)** | **n (%)** |
| Age | ≤ 30 |  |  |
|  | 31–40 |  | 6 (27%) |
|  | 41–50 |  | 7 (32%) |
|  | ≥51 |  | 7 (32%) |
| Gender | Female |  | 18 (82%) |
|  | Male |  | 4 (18%) |
| Site | Production-based |  | 16 (73%) |
|  | Office-based | 5 (16%) | 5 (23%) |
| Occupation | Officer | 8 (25%) | 3 (14%) |
|  | Nurse | 3 (9%) | 5 (23%) |
|  | Administration, management or quality service | 6 (19%) | 6 (27%) |
|  | Director | 2 (6%) | 0 (0%) |
|  | Teacher | 4 (13%) | 1 (5%) |
|  | Other (cleaner, kitchen staff, security, lawyer, economist, logistics department…) | 6 (19%) | 7 (32%) |
| Were they study staff (nurses, human resources, managers at sites…)? | Yes | 0 | 6 (27%) |
