# Supplementary material for: Implementation pilot study of community self-testing for COVID-19 among employees of manufacturing industries and their household members in 2022 to 2023
Source: PLOS Glob Public Health. 2024 Jun 5;4(6):e0003269. doi: 10.1371/journal.pgph.0003269 (PMC11152268; doi:10.1371/journal.pgph.0003269)
Supplement: Attachment — Submitted filename: 19112023 Review Selftest implementation.docx [file pgph.0003269.s012.docx]

**Implementation pilot study of community self-testing for COVID-19 among employees**

**of manufacturing industries and their household members in 2022 to 2023.**

This manuscript addresses a clear and important public health topic. The manuscript is well-written, and the implementation method is clear enough to allow replication in other settings. The findings are also well presented. However, the study has major and minor issues that need to be addressed by the authors before a publication is considered.

Below are concerns that we think need to be addressed the authors:

1. The manuscript’s abstract does not discuss the study results as presented in the result section, it rather focuses on diagnostic yield as if this was a diagnostic or a prevalence study. Findings from the pre-enrolment and the post-implementation interviews and those from the qualitative component should appear in this abstract.

I personally do not see the need of the following paragraph in the abstract: *“The study focused on five areas of the feasibility framework proposed by Bowen et al., including acceptability, demand, implementation, practicality and adaptation. The feasibility of an iterative an adaptive COVID-19 self-test delivery model in Malaysia was assessed, to deliver self-tests to the community via workplaces. FIND, together with the Ministry of Health Malaysia, Kedah State Health Department, Kuala Muda and Kulim District Health Offices, the Federation of Malaysian Manufacturers Kedah/Perlis Branch, and four industrial manufacturing companies, conducted a mixed-methods implementation pilot study between November 2022 and May 2023.”*

2. The first line of the manuscript reads: COVID-19 self-testing is essential for enabling individuals to perform self-care, screen themselves and, if they test positive, isolate themselves in a timely manner. Is “perform self-care” appropriate here?
3. If I understand well, the industries ‘employers were trained to perform self-tests and not their household members. Given the design of the study, the household members may have self-tested themselves without assistance from the household’s member trained for self-testing. Do we know how often this happened? What was the strategy used to minimise self-tests by untrained study participants?
4. In lines 202 and 203, it is clearly stated that no informed consent nor data were directly collected from households’ members while in Figure 1 last box, it is stated that 633 participants with complete information were included in the regression analysis for self-test in households’ members. In Table 2, the characteristics of these households ‘members are also presented. Did the authors get authorisation to analyse and publish data of study participants without their informed consent?
5. Is it possible to reorganise the flow diagram (Figure1) for it to account for all study participants including households ‘members reached in a comprehensive order? In its current state, I assume that the 39 participants who reported self-test results never completed the baseline and the post-implementation surveys.
6. From line 330 to line 343, the following words are regularly used “some changes, slight decrease, no significant changes” while the p values presented are always way below 0.05.
a. When using the above-mentioned terms, are you alluding to statistical or clinical differences? Because statistically, the differences are significant.
7. The data in table 4 is categorical and not continuous, why was t test used to assess differences?
8. As	As per the study flow chart (Fig 1), only 39 study participants reported self-testing results while in lines 361 to 365, it is reported that more than half of the study participants reported using self-tests during the study implementation.
a. Did many people report using self-test but not the test results? If yes, can you provide clear information? How many study participants reported using self-test without reporting the test results and how many reported using self-testing with results?
b. How many people reported negative and invalid self-test results?
c. After how long were the test results supposed to be reported to the study team? Results reported after a long period may have been affected (recall bias) and this should be discussed.
d. What was the process of reporting test results for household members?
9. In results presented from line to 378, interpretation of the odd ratios is more appropriate and would give a clear picture of the analysis. Some of the 95% CI include 1, please be cautious when interpreting the result.
10. What was the rational for using “Hosmer and Lemeshow test”?
11. In the qualitative results, is it possible to avoid terms like “more than half”, instead give the actual numbers e.g., 25/44(57%).
12. From line 505 to line 510, you present factors that may have affected willingness to report COVID-19 results and how this can be addressed, which I think is an important point. Why do you then compare findings from this study to low positive COVID-19 diagnostic reported in another study in Malaysia and to another study reporting high COVID-19 incidences (lines 510 to 514)? How do low or high COVID-19 diagnostics reported in these two studies link or justify a drop in willingness to report COVID-19 self-test results?
13. “**There was an increase in the percentage of participants who knew correctly use a nasal swab to collect a self-sample and how to interpret a positive result**”. Lines 520 and 521 need review.
14. Throughout the study I did not see where effective communication strategies were tested with a positive impact on self-test. In lines 545 and 546 read: “These findings highlight the importance of effective communications strategies for promoting public health interventions and ensuring accurate reporting of results for national surveillance”. Is this an assumption or finding from this study?
15. There is no discussion on the positive self-test results reported by the study’s participants and their households ‘members despite the statement in lines 522 and 523 that there was a decline in knowledge interpretation of the self-test result. There is a need to discuss the self-test results as they might be affected by many factors.
