# Supplementary material for: Implementation pilot study of community self-testing for COVID-19 among employees of manufacturing industries and their household members in 2022 to 2023
Source: PLOS Glob Public Health. 2024 Jun 5;4(6):e0003269. doi: 10.1371/journal.pgph.0003269 (PMC11152268; doi:10.1371/journal.pgph.0003269)
Supplement: Attachment — Submitted filename: Response letter_19112023 Review_12Jan24.docx [file pgph.0003269.s013.docx]

**Response to reviewers**

Reviewer #1:

Reviewer #1:

1-The message of the article is simple, and the performed study is demonstrative. I consider this manuscript useful for current practice in the field.

Authors’ response: Thank you for acknowledging the importance of our work.

Reviewer #1:

2- I think the introduction section can be improved a little bit. It would be better to show the current research progress in this field, so that the research gaps can be more clear. At present, I feel that the presentation of research gaps is a bit subjective.

Authors’ response: Thanks to the reviewer for this comment, we have broadened the literature review and included other research gaps in the operational and implementation research field within the Introduction. We have deleted some of the previous sentences to reduce the section and be able cite more publications.

Reviewer #1:

3- The process and methods are in general well described.

Authors’ response: Thank you for this comment.

Reviewer #1:

4- Please add more results from other research and compare your results with other authors' results

Authors’ response: Thanks for this comment. Even though there is scarce literature on the topic, we have reviewed the literature and included new publications in our Discussion section to compare our results with new publications.

Reviewer #1:

5- Please provide further information about the study limitation and future research.

Authors’ response: We have worked to improve the study limitations and future research information in the Discussion section (page 36 lines 629 to page 37, line 647).

Reviewer #1:

6- How did the researchers calculate the sample size?

Authors’ response: Thank you for the opportunity to clarify the methodology behind the sample size. As a mixed-methods implementation pilot, the primary objective was to assess the feasibility of the proposed model rather than to generalize the results to a larger population. Thus, the sample size was guided by practical considerations rather than statistical power calculations typically used in hypothesis-testing studies. This was an implementation study, without control group (due to ethical issues, as we aimed to allow all employees to self-test). We invited to participate in the study all the employees from the four sites. From the approximately 1500 potential participants from the sites, assuming a margin of error of 5%, a confidence level of 95% and a 50% response distribution, the minimum sample size of participants to complete the surveys was 306 participants. However, we expected a higher number of responses. As you can see in our results, we enrolled 1768 participants, 99% of them (1763) completed the baseline survey and 38% (674) responded to the post-implementation survey.

Reviewer #1:

7- How did the researcher ensure that the survey and the interview guide were valid and reliable?

Authors’ response: Thank you for giving us the opportunity to strengthen this issue. Before the study was implemented in the sites described, we conducted a piloting of the data collection tools in other manufacturing industry in Kedah state. All sites were selected by Kedah State Health Department. This was briefly detailed in the “Formative research” section of the “Methods” (page 7, line 173, to page 8 line 186), but have reviewed the text in the manuscript to be clearer on this topic.

Reviewer #1:

8- How did the researcher ensure the confidentiality of participants personal information?

Authors’ response: Thank you for your insightful comment regarding confidentiality. Ensuring the privacy and security of participants’ data was a key aspect of our study. In the “Potential Risks” section (page 13, lines 305-306) we included that “this risk was minimised by training study staff in ethics and confidentiality issues.” However, we have included more information in the Methods section to explain confidentiality issues within the section “Settings and participants” (page 9, lines 217 to 226), such as the inclusion of a unique identifier (study ID number) that was used for data collection for all study surveys.

Reviewer #1:

9- It would be advisable to elaborate more in the discussion regarding the quantitative phase results with comparison to the published studies in literature.

Authors’ response: Thanks for this comment. Even though there is scarce literature on the topic of COVID-19 self-testing implementation in workplaces, we have reviewed the literature and included new publications in our Discussion section to compare our results with recent publications.

Reviewer #2: **Implementation pilot study of community self-testing for COVID-19 among employees of manufacturing industries and their household members in 2022 to 2023.**

This manuscript addresses a clear and important public health topic. The manuscript is well-written, and the implementation method is clear enough to allow replication in other settings. The findings are also well presented. However, the study has major and minor issues that need to be addressed by the authors before a publication is considered.

Below are concerns that we think need to be addressed the authors:

Reviewer #2:

1. The manuscript’s abstract does not discuss the study results as presented in the result section, it rather focuses on diagnostic yield as if this was a diagnostic or a prevalence study. Findings from the pre-enrolment and the post-implementation interviews and those from the qualitative component should appear in this abstract.

I personally do not see the need of the following paragraph in the abstract: *“The study focused on five areas of the feasibility framework proposed by Bowen et al., including acceptability, demand, implementation, practicality and adaptation. The feasibility of an iterative an adaptive COVID-19 self-test delivery model in Malaysia was assessed, to deliver self-tests to the community via workplaces. FIND, together with the Ministry of Health Malaysia, Kedah State Health Department, Kuala Muda and Kulim District Health Offices, the Federation of Malaysian Manufacturers Kedah/Perlis Branch, and four industrial manufacturing companies, conducted a mixed-methods implementation pilot study between November 2022 and May 2023.”*

Authors’ response: Thank you very much for acknowledging the importance of our work. We have worked in this new version to address those major and minor issues. We have removed those sentences from the abstract and included more sentences with key results.

Reviewer #2:

2. The first line of the manuscript reads: COVID-19 self-testing is essential for enabling individuals to perform self-care, screen themselves and, if they test positive, isolate themselves in a timely manner. Is “perform self-care” appropriate here?

Authors’ response: Thanks for realising this. We have removed the word “perform” where not appropriate.

Reviewer #2:

3. If I understand well, the industries ‘employers were trained to perform self-tests and not their household members. Given the design of the study, the household members may have self-tested themselves without assistance from the household’s member trained for self-testing. Do we know how often this happened? What was the strategy used to minimise self-tests by untrained study participants?

Authors’ response: Thanks for giving us the opportunity to reflect on it. In July 2021, the MoH granted conditional approval to three brands of COVID-19 self-tests kits and as such COVID-19 self-tests started to be used by the public at that time. In August 2021, the MoH released a list of 11 brands that were also given conditional approval. This information is key to understand that people in Malaysia were not naive in the use of COVID-19 self-tests.

In our study, we provided trainings to study participants (staff at sites who signed the informed consent), to make sure they used correctly the specific self-tests chosen for the study and interpret their results. Study participants were advised to share this knowledge with their household members in case they would need to use one of the study self-tests. As reflected by the reviewer, we cannot guarantee that all study participants showed their household members how to self-test. However, for ethical reasons, our reasoning behind was to make the self-tests accessible and available to all household members.

Reviewer #2:

4. In lines 202 and 203, it is clearly stated that no informed consent nor data were directly collected from households’ members while in Figure 1 last box, it is stated that 633 participants with complete information were included in the regression analysis for self-test in households’ members. In Table 2, the characteristics of these households ‘members are also presented. Did the authors get authorisation to analyse and publish data of study participants without their informed consent?

Authors’ response: Sorry for the misunderstanding. We only collected information from participants (employees at sites who signed the informed consent). Participants reported “how many household members they had”, “if their family members/close friends have had COVID-19 disease”, and other indirect questions about their households. This data is presented in Table 2, as this was the baseline sociodemographic information collected by participants. At the end of the study, participants were asked “how many household members used a COVID-19 self-test" and “how many of them were positive”.

But we did not collect any information directly from household members. All the information collected regarding the households, was anonymized information given by the participants in the study regarding their households and aggregated numbers of tests used/number of household members with a positive result in last month's etc.

We have consent from the participants to publish all the data that has been gathered during the study, anonymously, via a unique identifier (study ID number). No personal identifiers were collected.

Reviewer #2:

5. Is it possible to reorganise the flow diagram (Figure1) for it to account for all study participants including households ‘members reached in a comprehensive order? In its current state, I assume that the 39 participants who reported self-test results never completed the baseline and the post-implementation surveys.

Authors’ response: Thanks again for such an important comment. We have reorganized the diagram to show the flow of participants better. It was challenging to represent the flow, as all participants were given the opportunity to respond to all surveys, whether they responded to the baseline survey or not, or responded to COVID-19 self-tests results via the results survey. We worked now to represent the flow in a more understandable way.

Reviewer #2:

6. From line 330 to line 343, the following words are regularly used “some changes, slight decrease, no significant changes” while the p values presented are always way below 0.05.
a. When using the above-mentioned terms, are you alluding to statistical or clinical differences? Because statistically, the differences are significant.

Authors’ response: Yes, we were mentioning statistically significant differences. Following the reviewers’ advice, we have removed the terms “slightly” and “some changes” to be more precise in our narrative. We have highlighted the statistically significant differences and the only value that was not statistically significant (p=0.24) was the willingness to use a self-test, we have clarified that in the results section.

Reviewer #2:

7. The data in table 4 is categorical and not continuous, why was t test used to assess differences?

Authors’ response: We appreciate your attention to this matter. In Table 4, the t-test serves as an appropriate method for assessing variations in agreement levels measured on a continuous 5-point scale. In the context of our analysis, where we are examining pre- and post-implementation changes, the Chi-square test would not be an appropriate method.

Reviewer #2:

8. As	As per the study flow chart (Fig 1), only 39 study participants reported self-testing results while in lines 361 to 365, it is reported that more than half of the study participants reported using self-tests during the study implementation.
a. Did many people report using self-test but not the test results? If yes, can you provide clear information? How many study participants reported using self-test without reporting the test results and how many reported using self-testing with results?
b. How many people reported negative and invalid self-test results?
c. After how long were the test results supposed to be reported to the study team? Results reported after a long period may have been affected (recall bias) and this should be discussed.
d. What was the process of reporting test results for household members?

Authors’ response: Thank you for the opportunity to explain this. We have worked in the manuscript text and in the flow figure to explain it better.

**Reporting survey (open to report results during the whole implementation):** Only 39 participants declared their self-test results during the implementation. These participants completed the survey a total of 57 times. The reporting survey was aimed to collect responses, at real time, every time a participant self-tested and wanted to report results. However, we were aware that participants might be reticent to report their results every time they self-tested (basically, for time constraints). We have clarified it in the sentence “An online reporting survey to disclose COVID-19 self-test results was available to be used throughout the entire implementation period (to report the number of tests used and their results)”.

**Post-implementation survey (to be used once, at the end of the implementation):** 674 participants responded to the post-implementation survey, where there were also questions to respond about their self-tests results. The post-implementation survey was aimed to collect knowledge, perceptions and experiences of the whole implementation, including questions regarding their use of the self-tests by them and their household members. From this survey, is from where we obtained these results, included within the “Uptake of COVID-19 self-tests" section of the results as “Of the 674 participants (38%) who completed the survey at the end of the study, more than half of participants (58.8%) and their household members (55.9%), as was reported by participants, used a COVID-19 self-test during the implementation (S6 Annex). In total, 40 participants and 100 household members reported they self-tested positive during the pilot study (S6 Annex).”

Answering point by point to your questions:

a. All the participants that answered the reporting and the post-implementation survey declared their self-test results as positive, negative, or invalid.
b. In the reporting survey, 39 participants reported a total of 57 times: **1 invalid result was reported (1,75% of all cases reported), and 54 negative results (94,7% of all cases reported)**. We have worked on the text where this survey was explained to make it more understandable (Results section, “Uptake of COVID-19 self-tests", page 28, lines 435-442). In the post-implementation survey, answered by 674 participants (Supplementary information Annex S6), **396 participants reported to have used at least a self-test,** **including 40 positive cases, 27 invalid cases and 329 negative cases**.
c. As briefly explained above, participants had the option to report results at any time, but most of them opted to report just at the end of the study. We acknowledged that there might be recall bias in their responses, as they might be answering questions regarding the last months up to a maximum of 7 months. We understand that the number of self-tests provided were limited, and that participants might have counted the self-tests remaining at home at the end of the study to know how many were used. Also, we understand that even though there might have been recall bias in the negative results, most participants might recall if they or their household members had COVID-19.
d. Household members were not participants in the study. They did not sign the informed consent; thus, we did not collect any data directly from them. However, as participants (staff at sites who signed the informed consent) had the opportunity to bring self-tests home to be used by their household members, we asked them how many self-tests were used by them, and the aggregated number of positive and invalid cases in their household.

We have worked on the methodology and on the flow to explain the study flow and the different data points in a clearer way for readers to understand the study.

Reviewer #2:

9. In results presented from line to 378, interpretation of the odd ratios is more appropriate and would give a clear picture of the analysis. Some of the 95% CI include 1, please be cautious when interpreting the result.

Authors’ response: Thank you for your suggestion. However, the analysis presented from line 381 onward specifically focuses on comparing knowledge about COVID-19 testing before and after the implementation, without considering any other variables. As a result, logistic regression was not applied. Instead, we opted for McNemar test to identify any observed differences. Logistic regression was only applied for COVID-19 testing uptake, with multiple variables were taken into consideration (line 394 and Table 4).

Reviewer #2:

10. What was the rational for using “Hosmer and Lemeshow test”?

Authors’ response: The Hosmer and Lemeshow test is a widely recognized method for validating logistic regression models. The test was employed as a means of assessing the goodness of fit for our logistic regression model. This test is particularly useful for evaluating whether or not the observed event rates match expected event rates in subgroups of the population, to determine how well our model predicts the outcomes for different groups within the data. It was a critical step in ensuring the robustness and reliabilitly of our logistic regression model.

Reviewer #2:

11. In the qualitative results, is it possible to avoid terms like “more than half”, instead give the actual numbers e.g., 25/44(57%).

Authors’ response: Thanks for this suggestion. We have removed those terms “most”, “more than half” etc. In line with best practices in qualitative methods our aim was not to quantify the qualitative results. Therefore, we are not providing numbers in the qualitative section of the manuscript. We have rewritten the text to include participants’ perceptions without quantifying them, as the key point of the qualitative section is participants “narrative”, their ideas, perceptions, and experiences, and not how many of them expressed that feeling or perception.

Reviewer #2:

12. From line 505 to line 510, you present factors that may have affected willingness to report COVID-19 results and how this can be addressed, which I think is an important point. Why do you then compare findings from this study to low positive COVID-19 diagnostic reported in another study in Malaysia and to another study reporting high COVID-19 incidences (lines 510 to 514)? How do low or high COVID-19 diagnostics reported in these two studies link or justify a drop in willingness to report COVID-19 self-test results?

Authors’ response: Thank you for realising this. We have included more sentences to explain better the publications whom we compare our results with. Also, we have broadened the literature review to reference other recent publications and compare our results with their results.

Reviewer #2:

13. “**There was an increase in the percentage of participants who knew correctly use a nasal swab to collect a self-sample and how to interpret a positive result**”. Lines 520 and 521 need review.

Authors’ response: Thanks for highlighting this. We have reviewed the sentence and changed it appropriately to read “There was an increase in the percentage of participants who correctly knew how to use a nasal swab to collect a self-sample and how to interpret a positive result.”

Reviewer #2:

14. Throughout the study I did not see where effective communication strategies were tested with a positive impact on self-test. In lines 545 and 546 read: “These findings highlight the importance of effective communications strategies for promoting public health interventions and ensuring accurate reporting of results for national surveillance”. Is this an assumption or finding from this study?

Authors’ response: Thank you for the opportunity to explain this. We agree with the reviewer that we did not include much information in the manuscript submitted. However, we conducted workshops with staff at sites and trainings with participants during the study. Different communication materials were used (posters, flyers, and continuous communication with the study research assistant that was at sites some days per week to solve questions etc.). We cannot state that our findings were related to a good communication strategy, but we can indicate that we provided a comprehensive package including different types of communications to potentially boost enrolment and increase adherence to self-testing and reporting. This was also covered in the qualitative section of the results, under Theme 3 were stated: “The training sessions also played a crucial role in providing individuals with the necessary guidance and instructions to perform self-testing effectively and correctly. Attending the training, one of the participants stated that “The training helped to understand how to use the self-test” (Female, 35 years old, executive). This highlights the positive impact training can have on promoting comprehension and proficiency in the use of self-tests.”

Reviewer #2:

15. There is no discussion on the positive self-test results reported by the study’s participants and their households ‘members despite the statement in lines 522 and 523 that there was a decline in knowledge interpretation of the self-test result. There is a need to discuss the self-test results as they might be affected by many factors.

Authors’ response: Thank you for bringing this important topic. We are aware that a faint line result causes misconceptions and doubts about its meaning in COVID-19 and other self-tests. Our results are especially relevant for manufacturers to make clearer instructions for use to explain potential end-users about the positivity of a faint line result, but also to program implementors to provide specific training, awareness, sensitization and continuous education to potential end-users to avoid misconceptions.

Our results showed a decline in knowledge regarding the interpretation of a faint result (from 58% to 48%). However, as the reviewer highlighted, this might be due to different factors. We have rephrased the sentence in the discussion: “However, there was a decline in knowledge regarding the interpretation of a faint-line result. This result might be attributed to various external factors beyond the scope of the study, including evolving public health messages, the dynamic nature of the epidemic, proliferation of COVID-19 information through press and social media, increased number of self-tests available in the market etc. which may have influenced participants’ understanding and perceptions of test results. This result is especially relevant for manufacturers to provide clearer instructions for use regarding the positivity of the faint line and to other program implementors to provide specific sensitization and continuous education. Further educational efforts should focus on clarifying the meaning of faint-line results and the appropriate actions individuals should take when encountering such results.”
