# Supplementary material for: Implementation pilot study of community self-testing for COVID-19 among employees of manufacturing industries and their household members in 2022 to 2023
Source: PLOS Glob Public Health. 2024 Jun 5;4(6):e0003269. doi: 10.1371/journal.pgph.0003269 (PMC11152268; doi:10.1371/journal.pgph.0003269)
Supplement: Attachment — Submitted filename: Response to reviewers.docx [file pgph.0003269.s014.docx]

**Manuscript PLOS GPH**

12^th^ of April 2024

PGPH-D-23-01835R1

Dear PLOS Global Public Health,

Thank you for considering our manuscript entitled: "Implementation pilot study of community self-testing for COVID-19 among employees of manufacturing industries and their household members in 2022 to 2023" for publication in PLOS GPH. Please see below our point-by-point responses to reviewer comments.

**Reviewer #2:** Implementation pilot study of community self-testing for COVID-19 among employees
of manufacturing industries and their household members in 2022 to 2023.
The manuscript has significantly improved compared to the first version but still has some minor issues that need to be addressed:
The abstract, though reviewed, still does not talk to the content of the paper:
1. None of the qualitative findings is clearly presented in the abstract. This is a mixed method, right?
2. The quantitative results are said to show significant differences and/or associations, but no statistical tests nor p-values are included in the abstract.

**Response to Reviewer #2:** Thank you very much for this comment. We have included in the abstract more information on the statistical tests performed and qualitative results.

Please see below issues that need to be addressed:
1. Line 38-39: Younger individuals (aged 30 and less), and males, showed a significant higher likelihood of self-testing compared to older and female participants.
Please add the test used to assess the association and the result (p value).
**Response to Reviewer #2:** Thank you so much for your valuable feedback. In response to your request we have added additional information in lines 38-39 as follows:

“Individuals aged 30 or less [adjusted odds ratio (AOR): 2.65; 95% CI: 1.63 to 4.31; p<0.001] and males (AOR: 1.54; 95% CI: 1.09 to 2.17; p= 0.014) showed a significant higher likelihood of self-testing compared to older and female participants”.

2. Line 40-41: Additionally, individuals who received three or more doses of a COVID-19 vaccine had higher odds of using self-tests.
Please add the odds results and 95% CI.
**Response to Reviewer #2:** Thank you for your feedback. To address your request for detailed information we have revised Lines 40-41 as follows: “Additionally, individuals who received three or more doses of a COVID-19 vaccine had higher odds of using self-tests (OR 1.56 (95% CI: 1.03 to 2.36, p = 0.037)).” We appreciate the opportunity to clarify this aspect of our findings further.

3. Line 41-42: Significant changes in participants’ perceptions and knowledge about
COVID-19 self-testing was noted.
Did you do any statistical significance test for change in knowledge? If no, please review this sentence and avoid “significant”.
**Response to Reviewer #2:** Thank you for your attention to detail and for highlighting the importance of clarity in our manuscript. In response to your comment regarding the use of the term "significant" in relation to changes in participants’ perceptions and knowledge about COVID-19 self-testing, we have reviewed and updated Lines 41-42 as follows: "There was a significant increase in participants’ knowledge on how to correctly collect a self-sample using a nasal swab from 36,9% at baseline to 43,6% post-implementation (p=0.004) and correct interpretation of a positive result from 80,5% at baseline to 87,6% post-implementation (p<0.001). Furthermore, there was a notable increase in the correct understanding of actions following a positive result, especially regarding self-isolation, which rose from 59.1% to 71.9% (p<0.001)."

4. Line 42-44: There was an increase in participants’ knowledge on how to correctly collect a self-sample using a nasal swab (from 36,9% to 43,6%) and interpret a positive result (from 80,5% to 87,6%).
Please add the test used to assess difference between 36.9% and 43.6%, and 80.5% and 87.6% and the results (p value). If you did not do statistical test, please avoid the term “significant”.
**Response to Reviewer #2:** Thank you for your comment. The revision specified above includes the statistical significance of the observed changes, supported by p-values from our analysis using the McNemar test. It reflects the improvement in knowledge and perceptions among participants following the self-testing program implementation. The test used was explained in the Methods section and appeared as Footnote in the Table where results where presented. We appreciate the reviewer’s guidance in enhancing the precision and clarity of our findings.

5. Line 209: “that” may need to be removed.

**Response to Reviewer #2:** Thank you so much for realizing this typo. We have removed it.

6. Line 222 to 223, please name the figure.

**Response to Reviewer #2:** The figure is named “Table 1. Timeline for data collection and implementation by site.”

7. Line 346 to 360, please specify test for each p value.

Response to Reviewer #2: Thank you for seeking clarification on the statistical tests associated with the p-values reported in Lines 346 to 360, relating to the changes in participants' perceptions of COVID-19 disease and self-testing before and after the pilot study implementation.

For each aspect described, a paired t-test was used to analyze the changes in mean scores derived from Likert scale responses from participants who completed both the baseline and post-implementation surveys. The choice of a paired t-test is appropriate for comparing the means at two different times, providing a robust analysis of changes in perceptions and attitudes. Below are the details corresponding to each mentioned change:

- Worry about COVID-19: A significant decrease in worry levels was observed, with the mean score changing from 3.89 (SD = 1.13) at baseline to 3.78 (SD = 1.07) post-implementation (p = 0.028; t = -2.20).
- Willingness to Perform a Self-Test: This was the only value that did not show a statistically significant change, with mean scores slightly decreasing from 4.26 (SD = 1.01) to 4.20 (SD = 0.96) (p = 0.243; t = -1.17).
- Willingness to Report a Self-Test Result: A significant decrease was noted in the willingness to report results, with the mean score changing from 4.40 (SD = 0.93) to 4.11 (SD = 1.03) (p < 0.001; t = -5.93).
- Understanding the Benefits of Self-Testing: Participants demonstrated a decrease in their understanding, with the mean score changing from 4.31 (SD = 0.88) to 4.11 (SD = 0.96) (p = 0.011; t = -2.56).

These analyses were conducted using the paired t-test, as noted in the footnote in Table 4, to evaluate the significant changes in perceptions and knowledge about COVID-19 self-testing among participants. The use of this test is substantiated by the data structure and the objective to assess mean differences over time within the same group.

We have updated those lines in the manuscript as follows: “The only value that was not statistically significant before and after the implementation was the willingness to perform a COVID-19 self-test, with the p-value indicating no significant change (p = 0.243) (Table 4). [...] Before the pilot study, 62.0% of participants strongly agreed and 23.7% agreed that they understood the benefits of self-testing; after the pilot study, these percentages changed to 51.2% and 34.7%, respectively (p=0.011) (Table 4).”

We hope this clarification adequately addresses your concerns and justifies the methodological approach taken in our analysis.

8. Table 4: I am still not convinced that t test is the appropriate test for the data in this table:
1. In the table, I see Mean (SD). What is the meaning of the mean? How did you calculated the mean using categorical data?
2. There are appropriate tests for agreement using categorical data. The data you have in this table is categorical.

**Response to Reviewer #2:** Thank you for your thoughtful inquiry regarding the use of the t-test in Table 4, which presents changes in perceptions of COVID-19 disease and self-testing. We realized that the presentation may have initially suggested a categorical analysis. However, the methodological approach is grounded in the following reasoning:

- **Mean Calculation and Data Nature:** The mean scores presented were calculated based on Likert scale responses, which, although discrete, can be treated as continuous for analysis purposes when the scale includes five or more levels. This approach is used in social sciences to evaluate changes or differences in perceptions and attitudes. Each response option from "Strongly disagree" (1) to "Strongly agree" (5) represents an ordinal position, which, when aggregated across respondents, yields a mean value indicative of the collective attitude or perception.
- **Paired t-test Justification:** The paired t-test was employed to compare the mean scores before and after the pilot study's implementation. This statistical test is appropriate when assessing the mean differences of the same group at two different times, particularly when the data distribution approximates normality for the differences or when the sample size is sufficiently large to invoke the central limit theorem.
- **Statistical Analysis of Ordinal Data:** Although the data originated from a Likert scale (ordinal), treating such data as interval for the purpose of applying a t-test is justified under the assumptions mentioned. This treatment allows for a nuanced analysis of shifts in perceptions, assuming the intervals between response options are considered equal.

There has been a longstanding debate regarding whether Likert scales can be converted to numerical values and treated as interval data. As per Suvillan et al. such conversion is permissible provided the data follows a normal distribution. (Sullivan GM, Artino AR Jr. "Analyzing and interpreting data from likert-type scales." J Grad Med Educ. 2013 Dec;5(4):541-2.)

We appreciate the opportunity to provide further clarification and ensure the robustness and transparency of our analytical methods. To clarify this methodological choice in the manuscript, we have added the following sentences in the Methods section including a brief description of treating Likert scale responses as continuous data under certain conditions, within the “Data analysis: Quantitative data” section of our manuscript: “The paired t-test was employed to compare the mean scores before and after pilot implementation. The mean scores were calculated based on Likert scale responses, that were represented as ordinal continuous data for analysis.”.

9. Line 365 to 371, for each p value please specify the test used.

**Response to Reviewer #2:** Thank you for your request for detailed clarification on the statistical tests used to derive the p-values mentioned in Lines 365 to 371 of our manuscript. The type of test performed (McNemar) is detailed in the Methods section and included as a Footnote to Table 5 in the Results section. To maintain conciseness and avoid redundancy, we chose not to repeat these details in the sentences, where the p-values are mentioned. We hope this approach meets the reviewer’s approval, but we remain open to revisit the text to include these details if it is believed that it would enhance the manuscript’s clarity. We appreciate the opportunity to clarify the statistical methods employed in our study and hope this addresses your concern.

10. Line 418-421: Among individuals of non-Malaysian origin, there was a significantly higher OR (9.05, 95% CI: 1.07, 76.52) of uptake of self-testing among their household members compared with Malaysians, indicating a strong association between country of origin and the use of self-tests (p = 0.043) (Table 7).
In this case I would not interpret the result as a strong association due to a very wide 95% CI and a p-value that is nearing 0,05. The wide 95% CI tells us that, the result is imprecise (it can as well be 1.08 instead of 9.05 or 76.4). This is because the number of non-Malaysians is very low in the study, and the result does not allow us to draw any conclusion.
Please be cautious when interpreting this result.
**Response to Reviewer #2:** Thank you for your valuable feedback on our interpretation of the association between country of origin and the uptake of self-testing among household members. We understand your concern regarding the broad 95% confidence interval (CI) for the odds ratio (OR) and the proximity of the p-value to 0.05, which indeed suggests a degree of uncertainty in the estimate. Reflecting on your insight, we recognize that the wide CI, ranging from 1.07 to 76.52, is indicative of a substantial variance in the estimate, largely attributable to the small sample size of non-Malaysian participants. This variation does introduce a level of imprecision that warrants a more cautious interpretation of the data. In light of your suggestion, we propose the following revision to Lines 418-421:

"Among individuals of non-Malaysian origin, there was a higher OR of 9.05 (95% CI: 1.07, 76.52) for the uptake of self-testing among their household members compared to Malaysians, suggesting a potential association between country of origin and the use of self-tests (p = 0.043) (Table 7). However, it is important to note that the wide 95% CI reflects considerable uncertainty around this estimate, cautioning against a definitive conclusion, given the small number of non-Malaysian participants, which likely contributed to the imprecision of the result."

This change acknowledges the limitations of the data and moderates the claim of a "strong association" to a "potential association," aligning with the statistical evidence and addressing reviewers’ concerns about overinterpretation. We appreciate your guidance in enhancing the accuracy and integrity of our findings.

11. Line 516-518 need review: “Participants in a similar self-testing program in Canada showed that 68% if tge 116 individuals that responded to the survey were very satisfied with the testing program and 31% were satisfied”.

**Response to Reviewer #2:** Thank you so much for realizing this. We have reworded the sentence as “In a similar self-testing program in Canada, 68% of the 116 respondents indicated that they were very satisfied with the testing program, and 31% were satisfied.”

Thank you so much for your valuable feedback that has been helpful to improve our manuscript.

Best regards,

Sonjelle Shilton (corresponding author)

**Reviewer #3:** The authors have addressed the comments of the previous reviewers.

**Response to Reviewer #3:** Thank you very much for appreciating that we have addressed all your comments. We believe that all reviewers’ comments have contributed to improving our manuscript.

Best regards,

Sonjelle Shilton (corresponding author)
